# Dataset of 15-minute values of active and reactive power consumption of 1000 households during single year

**DOI:** 10.1016/j.dib.2023.109588

**Published:** 2023-09-16

**Authors:** Matej Cenký, Jozef Bendík, Boris Cintula, Peter Janiga, Žaneta Eleschová, Anton Beláň

**Affiliations:** Slovak University of Technology in Bratislava, Institute of Power and Applied Electrical Engineering, Bratislava, Slovakia

**Keywords:** Power consumption, IMS, Active, Reactive, Energy, 15-minute

## Abstract

This article presents a comprehensive dataset comprising average 15-minute values of active and reactive energy consumption in 1000 anonymized households located in the Slovak Republic, a central European country, throughout the year 2016. The dataset provides a valuable resource for researchers and practitioners interested in analysing energy consumption patterns at the individual household level within the unique context of Central Europe. Privacy concerns are addressed through anonymization techniques, ensuring the dataset's compliance with ethical considerations and privacy regulations. However, ZIP code information is included for each household. Researchers can confidently analyze the data without compromising the households' confidentiality. The dataset offers significant opportunities for researchers to explore energy consumption patterns, develop targeted energy management strategies, and contribute to the advancement of sustainable energy practices.

Specifications Table

**Table:** 

Subject	Electrical and Electronic Engineering

Specific subject area	The dataset of average 15 minute values of active and reactive energy consumption of 1000 individual anonymized households, however information about ZIP and reserved capacity are public. Data are collected from a mix of rural and urban households, during year 2016.
Data format	Raw
Type of data	Collection of .json files (dataset with numbers and number arrays) and single .csv (numerical values)
Data collection	Data were collected using Intelligent measuring systems (IMS) power meters installed at Point of Consumptions of 1000 households. All households are situated in the Slovak Republic, ZIP code information is included. Data were provided for anonymous use and publishing by the local Distribution system operator (DSO).
Data source location	Institution: Slovak University of Technology in Bratislava, Institute of Power and Applied Electrical Engineering City/Town/Region: Bratislava, Ilkovičova 3 Country: Slovakia
Data accessibility	Repository name: Mendeley Data Data identification number: doi: 10.17632/pns69yxgrp.2 Direct URL to data: https://data.mendeley.com/datasets/pns69yxgrp/2

## 1 Value of the Data

• This data represents a random statistical selection of electrical energy consumption of 1000 households connected to LV network. Each household contains information about annual load shape curves of active and reactive power consumption with 15 min intervals. Typically, such data are inaccessible to the public. Also, the size of the statistical file represents a high added value to the dataset. Another added value of the dataset is information about the approximate geolocation of households.
• The dataset can benefit academics, researchers or anyone interested in the field of power engineering, energy efficiency or power consumption economic analysis. Data are helpful for anyone who works with modelling of power systems, power quality analysis, economic analysis and risk management.
• Data can be useful for modeling LV systems or to generate a probability models of power consumption. Such models can be further used for hosting capacities analysis of photovoltaic and electric vehicles integration into low voltage network. Size of dataset also allows to by useful in machine learning applications and studies.
• Largest benefit of the data is that values of power consumption are in 15 minute time steps. This time interval is normally used for billing of consumption. Another benefit is the information about lagging and leading reactive power.
• Loadshapes of active power consumption P and reactive power consumption Q can be used for typical load models in quasi -dynamic modelling used in softwares such as Matlab Simulink, OpenDSS, PSLF and others [1].

## 2 Data Description

The published data contain information about active and reactive(leading and lagging) power electricity consumption from randomly selected 1000 households in the Slovak Republic. Each household is a single point of consumption (PoC) and is connected to the low voltage (LV) network at a voltage level of 0.4 kV (phase to phase voltage). All PoC are anonymized, and the data do not contain any information about the consumers and their location. Each PoC has the identifier ``meterID'', which has a value of 1-1000 and is also indicated in the name of individual files [2].

Electricity consumption is shown in 15-minute intervals throughout the year 2016. Each 15-minute value is measured as an average active,P, and reactive,Q, power consumption in kW or kVar.

The dataset is published in raw form in .json format. The folder in the repository has the following structure. The folder contains 1000 .json files, each representing one PoC, one household.

There is also a file named "meter_info.csv" in the folder, which contains the ZIP code and reserved capacity of each PoC.

The following section describes the structure of .json files in detail. Each .json file contains the following columns:

• 'meterID' column: – identifier of point of consumption
• 'day' column: – date information, day in a year
• 'month' column: – date information, month in a year
• 'year' - date information, year
• 'lowConsumptionSum' – sum of the 15 minute values of active power consumption in current day between 00:00 -9:30 and 21:45 - 23:45. This is power consumption in low price tariff. [kWh/15min]
• 'highConsumptionSum' - sum of the 15 minute values active power consumption in current day between 9:45 - 21:30 This is consumption in high price tariff. [kWh/15min]
• 'maxConsumption' – maximum active power consumption in current day and measured 15 minute [kW]
• 'laggingReactivePowerSum' - sum of the 15 minute values of lagging reactive power consumption in a current day [kVar/15min]
• 'leadingReactivePowerSum' - sum of the 15 minute values of leading reactive power consumption in a current day [kVarh/15min]
• 'laggingReactivePower' – array of average 15 minute values of lagging reactive power starting from 00:00 up to 23:45. [kvar]
• 'leadingReactivePower' - array of average 15 minute values of leading reactive power starting from 00:00 up to 23:45. [kvar]
• 'consumption' - array of average 15 minute values of active power starting from 00:00 up to 23:45. [kW]

Rows of the .json file represent individual days of the year.

The following section describes the structure of meter_info.csv file in detail. The file contains the following columns:

• 'meterID' column: – identifier of point of consumption
• 'ZIP' column: – ZIP code for each point of consumption
• ' reservedCapacity' column: –reserved capacity for each point of consumption [kW]

**Fig. 1:**
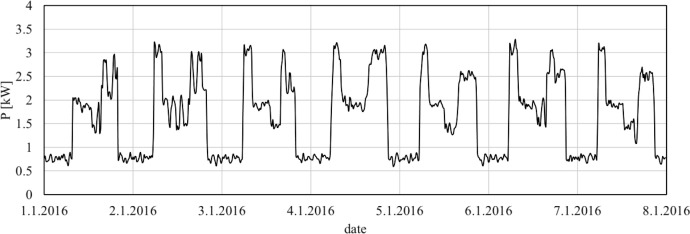
Example of data - active power consumption from the first to the 8th of January in PoC 104.

**Fig. 2:**
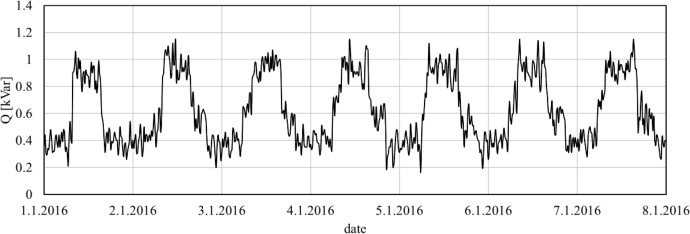
Example of data - lagging reactive power consumption from the first to the 8th of January in PoC 104.

The following graphs shows the active power consumption, Fig. 1, and lagging reactive power consumption, Fig. 2, from the first to the 8th of January in PoC 104.

## 3 Experimental Design, Materials and Methods

Presented power consumption datasets were acquired using industrial direct single phase or three phase power energy meters, intelligent measuring systems (IMS). All IMS power meter were property of distribution system operator and are used for continuous measurement of consumption and supply of active energy and reactive energy. The basic interval for remote reading and processing of measured data is at least ones per day. Specific brands and models of IMS are not known. In practice, IMS of various manufacturers are used. All IMS power meters were in accordance with Measuring Instruments Directive (MID) 2014/32/EU [3]. The accuracy class for all used IMS power meters for measuring active and reactive power consumption was 1 in accordance with the IEC 62053-21:2003 [4].

DSO agreed to publish the approximate location of installed IMS devices (ZIP code level). Information on the reserved capacity of PoC was also provided. These are published as the maximum consumption allowed of PoC in KW. Further details on IMS devices and PoC parameters are not available for publication.

The data provided are in the form of .json files. We recommend using the simple Python script below for data processing.



Another option how to quickly work with data are online converters [5].

## Limitations

Not applicable.

## Ethics Statement

This dataset does not require ethical approval, as the data on the energy consumption of households was obtained with the consent of the distribution system operator and have undergone anonymization.

## CRediT authorship contribution statement

**Matej Cenký:** Conceptualization, Writing – original draft. **Jozef Bendík:** Writing – review & editing. **Boris Cintula:** Resources. **Peter Janiga:** Supervision. **Žaneta Eleschová:** Funding acquisition. **Anton Beláň:** .

## Data Availability

Dataset of 15-minute values of active and reactive power consumption of 1000 households during single year (Original data) (Mendeley Data).
